# Is pelvic ultrasound useful in the clinical assessment and management of women with right iliac fossa pain? A single-centre retrospective study

**DOI:** 10.1308/rcsann.2023.0098

**Published:** 2024-04-05

**Authors:** HR Standing, KF Boag, EC Hamstead, SR Vaughan-Williams, MT Hughes, ABP Peckham-Cooper

**Affiliations:** Leeds Teaching Hospitals NHS Trust, UK

**Keywords:** Pelvic, Ultrasound, Useful, Clinical, Management

## Abstract

**Introduction:**

Acute right iliac fossa (RIF) pain is a common presenting symptom in surgical patients, with a wide range of differentials, particularly in premenopausal females. This study explores ultrasound usage in the management of women aged 16–55 years presenting with RIF pain.

**Methods:**

A total of 1,082 patients who presented to a tertiary hospital over 12 months were included. Data were collected from patients' electronic records, including initial clinical impression, imaging, management, operative findings, histology and subsequent hospital attendances within 6 weeks and within 6 months.

**Results:**

Following clinical assessment, 607 (56%) of patients underwent an ultrasound. Of these, 280 (25.9%) patients received no radiological imaging on initial presentation, and 252 (42%) had pathology identified on ultrasound. The most common finding was an ovarian cyst, closely followed by unexplained free pelvic fluid. Of the 607 patients scanned, 29 (4.8%) had an ultrasound diagnosis of appendicitis; 254 of 1,082 (23.5%) patients underwent operative management. Of the 254 patients who had surgery, 179 (70.5%) had preoperative imaging. Of the 29 (11.4%) cases where the intraoperative finding was gynaecological, 15 (51.7%) cases had not had any preoperative imaging. The negative appendicectomy rate was 21.3% (45/211). Of the 45 patients who had a histologically normal appendix, 22 (48.9%) had not had any previous imaging. Ultrasound had a specificity of 78% for diagnosing appendicitis.

**Conclusions:**

In patients who underwent operative management, a negative finding or finding not requiring surgical management was associated with no preoperative imaging. This supports the use of ultrasound scans as an adjunct in a multimodal approach to the assessment of women presenting with RIF pain.

## Introduction

Acute right iliac fossa (RIF) pain is a common presenting symptom in surgical patients, with a wide range of differentials. RIF pain can be primarily surgical, gynaecological and urological in origin. Diagnosis can be challenging in premenopausal females, where there can be difficulty in distinguishing acute appendicitis from an alternative pathology.

Ultrasonography, abdominal computed tomography (CT) and magnetic resonance imaging (MRI) are the imaging modalities used most commonly where there is diagnostic uncertainty.^1^ Data from the RIFT study by the West Midlands Research Collaborative, 2020, reported that the rate of negative appendicectomies performed in the UK is 20.0%, and demonstrated that women were more than two times as likely to have a negative appendicectomy compared with men, with the negative appendicectomy rate in women aged 16–45 years being 28.2%, versus 12.1% in men.^2^ The UK has one of the highest rates of negative appendicectomies.^3–5^ Postoperative complications following a laparoscopic appendicectomy include small bowel obstruction, superficial wound infection, intra-abdominal sepsis, stump leakage and stump appendicitis^6,7^; therefore, risks associated with performing appendicectomy are not insignificant and a reduction in the negative appendicectomy rate could reduce patient morbidity, reducing hospital admissions and the financial implications associated with this group.

The evidence is mixed in relation to the value of ultrasound in suspected appendicitis. The benefits of ultrasound are that it uses no radiation or contrast. Ultrasound is also a relatively quick and inexpensive imaging modality that can be a useful adjunct to the diagnostic work-up when there is clinical uncertainty. Limitations of ultrasound in the diagnosis of appendicitis include that the scan relies on the skill of the ultrasound technologist, more so than CT, and that bowel loops can obscure the appendix from being visualised.^8^ Ultrasound has also been shown in some studies to have low sensitivity (0.36) for appendicitis,^2^ but is useful in identifying gynaecological pathology. CT scanning, on the other hand, has high sensitivity and specificity in the diagnosis of appendicitis; however, it is used with caution particularly among females of childbearing age due to concerns over the effect of radiation on reproductive organs and cumulative cancer risk.^9^

The WSES Jerusalem 2020 Guidelines for the diagnosis and treatment of acute appendicitis recommend “the routine use of a combination of clinical parameters and US to improve diagnostic sensitivity and specificity and reduce the need for CT scan in the diagnosis of acute appendicitis”,^6,7^ and acknowledge low-dose CT imaging as an adjunct to clinical scoring systems and point-of-care ultrasound where there is clinical uncertainty in adult patients.^6,7^

There is no consensus as to how and when ultrasound scanning should be used in suspected appendicitis. This study explores the use of ultrasound in the management of female patients aged 16–55 years presenting acutely with RIF pain.

## Aims

The aims for this study are to (i) determine whether ultrasound imaging has had an impact on the management of premenopausal women presenting with RIF pain and (ii) assess the impact of ultrasound imaging on the likelihood of patients re-presenting to hospital acutely if managed nonoperatively in the first instance.

## Methods

### Data collection and eligibility

Patients were identified retrospectively by reviewing electronic acute surgical handover lists over a 12-month period (1 March 2019–29 February 2020 inclusive) from a large tertiary centre hospital in the UK. A new handover list is initiated 24-hourly following the 8am handover, is updated throughout the day and includes a discharge section for patients sent home on the same day. Inclusion criteria:

• Birth gender, female;
• 16–55 years old at presentation;
• Attendance to surgical assessment unit;
• Presenting complaint of RIF pain.

All eligible patients’ electronic records were reviewed. All included patients were referred either from their general practitioner or the Emergency Department for further surgical assessment. All patients were assessed by a surgical junior and registrar, and had imaging arranged as clinically indicated. Patients who remained in the hospital would be reviewed on the Consultant post-take ward round daily. Data were inputted on a Microsoft Excel spreadsheet, with a dropdown menu in each data category to validate entries and facilitate analysis. Several reviewers were involved in the collection of data, and an additional random sample was taken and crosschecked by the lead author to ensure reliability. Any data entries of uncertainty were resolved between the data collection team, or escalated to the senior project lead. Patients with incomplete datasets and duplicate patient entries were excluded.

These data were subsequently synthesised using the data sorting and analysis functions in Excel, which was undertaken by two members of the team separately to ensure reliability; the results are presented graphically.

### Baseline demographics

Baseline data included age, initial impression following assessment by a surgeon, type of imaging, imaging results, management, intraoperative findings, histology and any subsequent reattendances to hospital within 6 weeks and within 6 months.

### Ultrasound findings

Specifically for ultrasound, the results of the scan were collected verbatim. During analysis, findings of a similar nature were grouped together to simplify the presentation of the results. ‘Uterine pathology’ includes fibroids, uterine polyps, endometrial thickening, adenomyosis and endometriosis. ‘Biliary pathology’ includes gallstones, gallbladder sludge, cholecystitis and dilation of the common bile duct. ‘Bowel pathology’ includes terminal ileitis, dilated loop of bowel, bowel wall thickening and faecal loading.

### Intraoperative findings and histology

Intraoperative findings and histology report were recorded verbatim, and whether the procedure was a ‘positive’ or ‘negative’ appendicectomy was noted. A positive appendicectomy was defined with histology confirming appendicitis or where an intraoperative diagnosis was perforated appendicitis and histology was not obtainable. A negative appendicectomy is the removal of a histologically normal appendix. Sensitivity and specificity for ultrasound in diagnosing appendicitis were calculated.

### Unplanned patient return

Where patients had unplanned returns to hospital, the reason for presentation and the outcome of the attendance was recorded. Their initial attendance imaging and management was scrutinised.

## Results

### Baseline demographics and imaging

A total of 1,082 patients were included for the study. Of the eligible patients, 22 were excluded (*n*=4 duplicate entries, *n*=18 due to incomplete data). Age ranged from 16 to 55 years with a mean of 30 years. For further information on baseline demographics, see Table 1.

**Table 1:** Number of patients in each age category, and the mean, median, mode and standard deviation of patient ages

Age (years)	16–20	21–25	26–30	31–35	36–40	41–45	46–50	51–55	16–55
Frequency (*N*)	209	235	189	152	105	82	58	52	1,082
Mean (years)	30								
Median (years)	28								
Mode (years)	21								
Standard deviation (years)	10.2								

After initial clinical assessment, 802 patients (74%) had imaging. Of the 1,082 patients, 607 (56%) underwent a pelvic ultrasound scan (transabdominal and/or transvaginal approach); 95 of these patients went on to have a CT scan. Indications for the patients who had only an x-ray and no ultrasound included bowel obstruction, colitis, foreign body ingestion (lithium battery), renal calculi and complication of inflammatory bowel disease.

A total of 475 patients did not have an ultrasound scan; 173 (36.4%) had a CT scan alone, 18 (3.8%) an abdominal x-ray and 4 (0.8%) an MRI scan. Of the 1,082 patients, 280 (25.9%) received no radiological imaging on their initial presentation.

### Ultrasound findings

**Figure 1:**
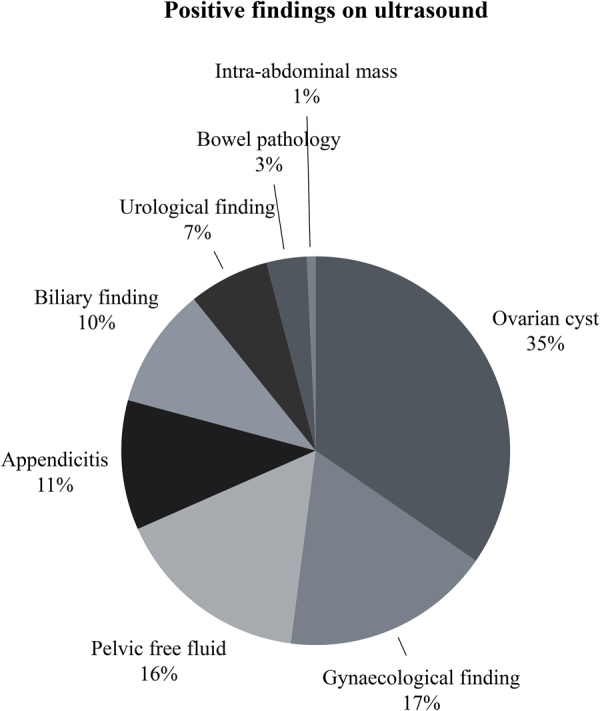
Pie chart demonstrating positive ultrasound findings (total of 269 findings)

Of the 607 patients scanned, 252 (42%) had a positive finding (pathology identified) on ultrasound, and 355/607 (58%) had no pathology on ultrasound. Some patients had more than one pathology identified on ultrasound, resulting in 269 positive findings on 252 patients. The positive ultrasound findings are demonstrated in Figure 1 and Table 2, with the most common finding being an ovarian cyst in 93 cases (15.3%), followed by unexplained free fluid in the pelvis in 44 (7.2%) cases; 31 (5.1%) patients had an ultrasound diagnosis of appendicitis. Overall, 140 (23.1%) patients had an ultrasound demonstrating a gynaecological diagnosis (including ovarian torsion, ovarian cyst, pregnancy and pelvic inflammatory disease).

**Table 2:** Frequency of each positive ultrasound finding

Ultrasound finding	Frequency (*N*)
Ovarian cyst	93
Gynaecological finding	47
Pelvic free fluid	44
Appendicitis	29
Biliary finding	27
Urological finding	18
Bowel pathology	9
Intra-abdominal mass	2

### Intraoperative findings and histology

Of the 1,082 patients, 254 (23.5%) underwent operative management of their condition; of these, 250 underwent a diagnostic laparoscopy.

Of the 254 patients who had surgery, 179 (70.5%) had preoperative imaging, 94 (37%) ultrasound, 63 (24.8%) CT, 18 (7.1%) both ultrasound and CT, 1 (0.4%) MRI, 3 (1.2%) abdominal x-ray, and 75 (29.5%) had no imaging preoperatively.

**Figure 2:**
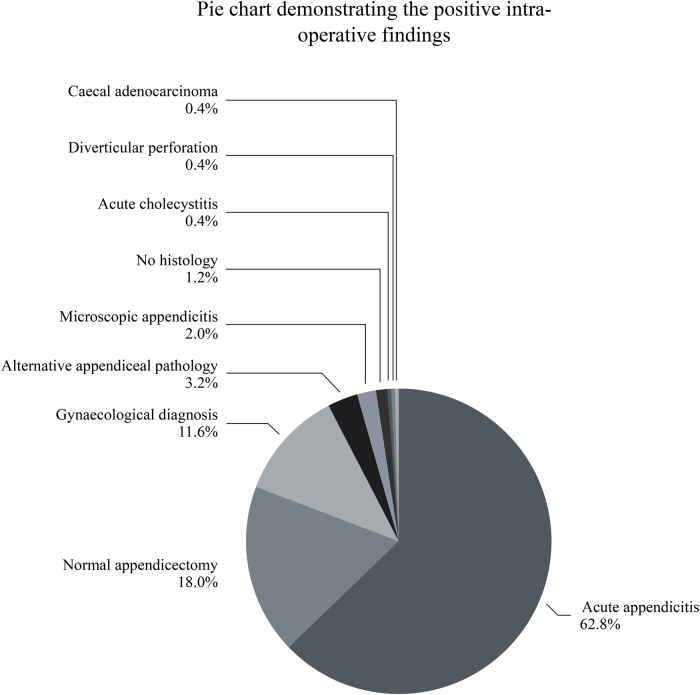
Pie chart demonstrating intraoperative findings in patients who underwent a diagnostic laparoscopy

The laparoscopic intraoperative findings are demonstrated in Figure 2. In five cases (2%), no pathology was identified intraoperatively and the appendix was left in situ, recorded as a negative diagnostic laparoscopy. In 211 (84%) cases, an appendicectomy was performed.

A histologically normal appendix was removed in 45 of the 211 cases; therefore, the negative appendicectomy rate was 21.3%. Histology confirmed 157 cases of acute appendicitis. In nine cases an alternative appendiceal/caecal pathology was identified; three low-grade appendiceal mucinous neoplasms, two cases of appendiceal enterobius vermicularis, two cases of Crohn’s disease, one goblet-cell carcinoid tumour and one caecal adenocarcinoma. Of the 157 appendicitis cases, 5 (3.2%) were microscopic, appearing normal intraoperatively. In three cases, histology results were not available, two as there were no histopathology specimens, due to perforated appendicitis that was managed with washout and drain insertion. In the third case, it is unclear why there was no histopathology result from the laboratory.

Of the 29 cases where the intraoperative finding was gynaecological in nature, 15 (51.7%) had not had any preoperative imaging. Of the 45 patients who had a histologically normal appendix, 22 (48.9%) had not had previous imaging.

### Sensitivity and specificity of ultrasound in diagnosing appendicitis

**Figure 3:**
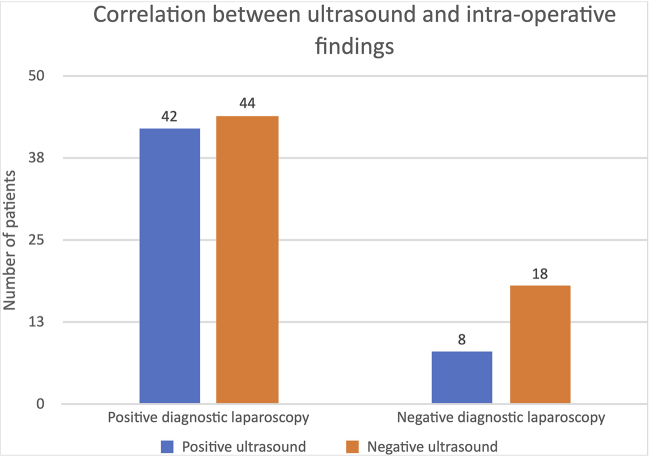
Bar chart showing correlation between ultrasound and intraoperative findings, in total of 112 patients who underwent ultrasound before surgery

In 24 cases, appendicitis was diagnosed on ultrasound and the intraoperative findings confirmed this – a true positive value of 24. In five cases, appendicitis was diagnosed on ultrasound but intraoperatively a normal appendix was found – a false positive value of 5 (Table 3). In 42 cases, ultrasound did not diagnose appendicitis, and intraoperatively appendicitis was diagnosed – a false negative value of 42. In 47 cases, ultrasound did not diagnose appendicitis and intraoperatively a normal appendix was identified – a true negative value of 47. Therefore, the sensitivity for ultrasound in diagnosing appendicitis in this study was 36% (24/24+42), and the specificity was 78% (47/47+5) (Figure 3).

**Table 3:** Ultrasound and intraoperative findings in a total of 112 patients who underwent ultrasound before surgery

	Ultrasound: appendicitis	Ultrasound: not appendicitis
Intraoperatively: appendicitis	24	42
Intraoperatively: not appendicitis	5	47

### Unplanned patient return

A total of 196 (18.1%) patients returned to the surgical assessment unit within 6 months of their initial attendance; 124 (63.3%) presented within 6 weeks. Of these 196 patients, 34 (17%) were postoperative patients; 112 (57.1%) presented with the same symptoms and were seen again by the general surgery, urology, gynaecology or gastroenterology team, whereas 50 (25.5%) presented with different symptoms.

Of the 196 patients who returned, 70 (35.7%) had not had an ultrasound on initial presentation, and 38 (19.4%) had had no radiological imaging on initial presentation.

**Figure 4:**
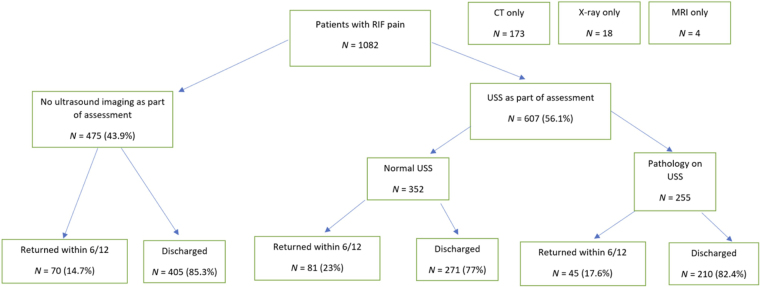
Flow chart showing proportion of patients scanned, positively diagnosed, discharged and returned within 6 months

Of the 112 who re-presented with the same symptoms, 32 underwent an ultrasound scan on their return attendance, 23 (71.9%) of which showed no abnormality. Of these 112 patients, 16 had a CT scan, 11 of which showed no abnormality (68.8%) (see Figure 4 for further details).

Of the 475 patients who did not have an ultrasound scan on initial presentation, 70 (14.7%) returned. Of these 70 patients, 44 had had a CT on initial presentation. Of the 607 patients who did have an ultrasound scan at initial assessment, 126 patients returned (20.8%).

## Discussion

In patients who underwent ultrasonography, the most commonly identified pathology was gynaecological in nature, making up over half of the positive ultrasound findings. The primary management of the majority of these gynaecological pathologies is nonoperative. Therefore, an ultrasound diagnosis of a nonoperatively managed gynaecological pathology prevents such patients being taken to theatre for a diagnostic laparoscopy. This is supported by the results showing that 15 out of 29 (51.7%) patients found to have a gynaecological pathology intraoperatively had not undergone preoperative imaging, as compared with the overall proportion of patients who were operated on who did not have any preoperative imaging, which was 75 of 254 (29.5%).

It is important to acknowledge that, although a relatively high proportion of gynaecological findings were identified on ultrasound, in some cases these may have been incidental or physiological in nature, and would not necessarily have explained the patient's symptoms. A limitation of ultrasound scans is that they cannot consistently correctly identify pathology, as demonstrated by their reasonably low sensitivity for detecting appendicitis in this study centre, at 36%. The results of this study show that the specificity of ultrasound for detecting appendicitis is better at 78%, which indicates they are of some use in ruling out pathology, and may be of value when used in conjunction with other tests and clinical examination, but the results of an ultrasound should not be relied on in isolation when making a diagnosis.

There are limitations in the use and application of ultrasonography; it relies on the skills of the ultrasonographer in scanning and diagnosis, leading to a larger margin of human error, which may lead to missed or incorrect diagnoses being made. Ultrasonography is also limited by practical elements such as bowel gas obscuring views, and challenges in patients of a larger body habitus. Additionally, despite ultrasounds being relatively quick and cheap, some patients experience delays in receiving ultrasound scans, and they are not readily available out of hours, which could delay operative management of a patient with appendicitis.

A meta-analysis from 2018 highlights the important distinction between uncomplicated and complicated appendicitis, and that, in suspected uncomplicated appendicitis, a delay of surgery for up to 24h from admission is not associated with a statistically significant increase in complications such as perforation.^10^ In complicated appendicitis, surgical delay is not advocated. In addition, there is increasing interest in the conservative management of appendicitis with antibiotics—an approach utilised during the COVID-19 pandemic, with some studies supporting that primary antibiotic treatment has the same outcomes at 90 days as acute appendicectomy in cases of appendicitis where there is no faecolith present.^10^

The female negative appendicectomy rate from this data is 21.3% (45/211), which is an improvement from the 2020 RIFT Study Group, with a negative appendicectomy rate of 28.2% in women.^2^ Of the patients who had a negative appendicectomy or had negative findings on diagnostic laparoscopy, 51.2% did not have any preoperative scans performed. This is a larger proportion of patients when compared with the figure of 29.5% of patients overall who were operated on but not scanned preoperatively. The fact that a larger proportion of the patients who were not scanned preoperatively had negative appendicectomies and negative laparoscopies suggests that a preoperative scan may be of some use in preventing such operations from taking place and/or supporting the decision to operate.

There will inevitably still be negative appendicectomies and negative laparoscopies that take place, unless all patients underwent preoperative CT scanning, but these figures indicate that the occurrence of such events could be reduced by preoperative ultrasound scanning. With the relatively good specificity of ultrasound, as mentioned above, a negative ultrasound may have prevented some operations being undertaken in such patients. This would avoid subsequently exposing such patients to the short- and long-term morbidity associated with a diagnostic laparoscopy.^11^

Our results demonstrated that 34 of the 250 patients (13.6%) who had diagnostic laparoscopies returned with postoperative complications within 6 months, ranging from wound infections to intra-abdominal collections, which demonstrates the potential morbidity of surgery. In addition to the impact on individual patients, a reduction in the number of negative appendicectomies and negative diagnostic laparoscopies could offer benefits locally and to the wider NHS, by enabling theatres to run more efficiently, increasing cost effectiveness and being more environmentally friendly by reducing unwarranted operations and the resources used to facilitate this.

The returns rate was lower in the nonultrasound-scanned group, at 14.7% (70/475) as opposed to the 23% (81/352) in the group who had normal ultrasound scans and the 17.6% (45/255) in the ultrasound-scanned group with pathology identified. It is not possible to establish the explanation for this pattern, but it is likely to be multifactorial. It is likely that patients who were not scanned were more well clinically or were deemed at lower risk, and therefore had less significant pathology that would be more likely to self-resolve. In this case, such patients would be less likely to return.

It would be interesting to explore whether there is any psychological reassurance or increased anxiety for patients following a normal ultrasound scan, as our results show that the patients in the normal ultrasound category have the highest likelihood of returning. In the patients who returned with the same symptoms and had an ultrasound scan on this attendance, 71.9% (23/32) of ultrasounds were reported as no abnormality detected.

Some weaknesses in this study are that it looked at data from only a single centre, which means the findings may not be generalisable across multiple centres. There will be differences between hospitals and trusts with regards to local protocols and accessibility of ultrasonography that would likely have an impact on the way such patients are investigated and managed. These data were collected, in part, during the COVID-19 pandemic, during which there were some changes to the approach of managing suspected appendicitis. In general there was a higher threshold to justify operating on patients due to the multitude of challenges faced, including the increased risks intraoperatively and postoperatively in the presence of COVID-19 infection, staffing levels and widespread pressure on hospital beds. The retrospective nature of the data, and reliance on handover sheets for data collection is also a weakness, as patients may have been missed from the study due to unclear/absent documentation.

## Conclusion

Ultrasound identified pathology in 42% of patients, with over half of positive findings being primarily gynaecological in origin. In patients who underwent operative management, a negative finding or finding that did not require surgical management was associated with no preoperative imaging being performed. Ultrasound had a specificity of 78% for appendicitis in this study, which supports the use of ultrasound scan to exclude appendicitis when used as an adjunct in initial assessment. However, ultrasound should not be relied on solely for diagnostic purposes due to its low sensitivity, and a multimodal approach, including blood tests, imaging and clinical assessment, remains the recommendation and standard of care for this patient cohort.

In conclusion, any female patient who is not postmenopausal who presents with RIF pain and who does not have a definitive diagnosis following history taking and examination should be considered for an ultrasound, unless the most likely differential diagnosis is one for which a CT scan would be the preferred diagnostic modality. Additionally, any female patient booked for diagnostic laparoscopy, where clinical parameters and time to theatre allow, should have an ultrasound scan. This approach, although increasing the radiological workload, will decrease the number of nontherapeutic surgical procedures undertaken on patients and reduce morbidity.

This paper is not based on a previous communication with a society or meeting.

The data utilised in this paper is available upon request.
